# Structure of the Bacterial Cellulose Ribbon and Its Assembly-Guiding Cytoskeleton by Electron Cryotomography

**DOI:** 10.1128/JB.00371-20

**Published:** 2021-01-11

**Authors:** William J. Nicolas, Debnath Ghosal, Elitza I. Tocheva, Elliot M. Meyerowitz, Grant J. Jensen

**Affiliations:** a Division of Biology and Biological Engineering, California Institute of Technology, Pasadena, California, USA; b Howard Hughes Medical Institute, Pasadena, California, USA; Université de Montréal

**Keywords:** cellulose, *Gluconacetobacter*, electron cryotomography

## Abstract

Cellulose is a widespread component of bacterial biofilms, where its properties of exceptional water retention, high tensile strength, and stiffness prevent dehydration and mechanical disruption of the biofilm. Bacteria in the genus *Gluconacetobacter* secrete crystalline cellulose, with a structure very similar to that found in plant cell walls. How this higher-order structure is produced is poorly understood. We used cryo-electron tomography and focused-ion-beam milling of native bacterial biofilms to image cellulose-synthesizing Gluconacetobacter hansenii and Gluconacetobacter xylinus bacteria in a frozen-hydrated, near-native state. We confirm previous results suggesting that cellulose crystallization occurs serially following its secretion along one side of the cell, leading to a cellulose ribbon that can reach several micrometers in length and combine with ribbons from other cells to form a robust biofilm matrix. We were able to take direct measurements in a near-native state of the cellulose sheets. Our results also reveal a novel cytoskeletal structure, which we have named the cortical belt, adjacent to the inner membrane and underlying the sites where cellulose is seen emerging from the cell. We found that this structure is not present in other cellulose-synthesizing bacterial species, Agrobacterium tumefaciens and Escherichia coli 1094, which do not produce organized cellulose ribbons. We therefore propose that the cortical belt holds the cellulose synthase complexes in a line to form higher-order cellulose structures, such as sheets and ribbons.

**IMPORTANCE** This work’s relevance for the microbiology community is twofold. It delivers for the first time high-resolution near-native snapshots of *Gluconacetobacter* spp. (previously *Komagataeibacter* spp.) in the process of cellulose ribbon synthesis, in their native biofilm environment. It puts forward a noncharacterized cytoskeleton element associated with the side of the cell where the cellulose synthesis occurs. This represents a step forward in the understanding of the cell-guided process of crystalline cellulose synthesis, studied specifically in the *Gluconacetobacter* genus and still not fully understood. Additionally, our successful attempt to use cryo-focused-ion-beam milling through biofilms to image the cells in their native environment will drive the community to use this tool for the morphological characterization of other studied biofilms.

## Abstract (precis)

This work’s relevance for the microbiology community is twofold. It delivers for the first time high-resolution near-native snapshots of *Gluconacetobacter* spp. (previously *Komagataeibacter* spp.) in the process of cellulose ribbon synthesis, in their native biofilm environment.

## INTRODUCTION

Humans rely on cellulose for building material, clothing, and fuel (1–3). Recently, the polymer has sparked interest in the biotechnology field as a potential source of biofuel feedstock (4) and in the biomedical industry as a biologically neutral scaffold to promote tissue regeneration (5, 6). Cellulose is a linear polymer of glucose molecules connected with β-1,4 linkages by a glucosyltransferase. Individual linear glucan chains can pack via hydrogen bonding and van der Waals interactions in various ways to form different types of celluloses, with different properties (3, 7, 8). The most common way glucan chains organize in nature is to form hydrogen-bonded planes stacked into parallel layers via van der Waals interactions (9, 10). These stacked layers give rise to cellulose I microfibrils, or native cellulose, that can then coalesce to form larger arrays. Because glucan chains pack in a regular lattice but cannot sustain this regular pattern over their entire length, cellulose I is considered paracrystalline. Depending on how the lattice is organized, cellulose I can be of the α form, bearing a triclinic unit cell, or β form, bearing a monoclinic unit cell (11, 12). Cellulose Iβ is mainly found in plants, where it is a major structural element of the cell wall (13).

In the prokaryotic world, cellulose is an important component of bacterial biofilms (14, 15), which increase cells’ tolerance for a range of biotic and abiotic stresses and enhance surface adhesion, cell cooperation, and resource capture (14). Cellulose-containing biofilms have also been shown to be involved in pathogenicity, enabling bacteria to resist antibiotics and disinfection (16, 17). Most cellulose-synthesizing bacteria produce amorphous (noncrystalline) cellulose, but a few genera, including *Gluconacetobacter*, can produce cellulose Iα microfibrils. In *Gluconacetobacter*, these paracrystalline cellulose microfibrils can further aggregate into wide ribbon structures and larger arrays (18), giving rise to thick biofilms that are predominantly pure cellulose I.

Bacterial cellulose is synthesized by an envelope-spanning machinery called the bacterial cellulose synthase (BCS) complex, encoded by the BCS gene cluster and first identified in *Gluconacetobacter* (15). While the components vary, most of the species encode BcsA, a component in the inner membrane that, with BcsB, catalyzes transfer of UDP-glucose to the nascent glucan chain (15, 19, 20). BcsD forms a periplasmic ring thought to gather glucan chains from several BcsA/B units (21, 22). BcsA and BcsB are essential for cellulose synthesis *in vivo*, and BcsD is essential for the crystallization of nascent glucan chains (23). BcsC forms a pore in the outer membrane (OM), and very recent work has solved its crystallographic structure (24). Consistent with previous data relying on sequence homology with the exopolysaccharide secretin components AlgE and AlgK from Pseudomonas aeruginosa, BcsC is found to form an outer-membrane β-barrel pore at its C-terminal end, secreting the nascent elementary cellulose fibrils into the environment (23–27). It is hypothesized that the elementary cellulose fibrils can aggregate with neighboring elementary fibrils upon secretion to form microfibrils (28, 29). Genes flanking the operon, *cmcAx* (endo-β-1,4-glucanase), *ccpAx* (unknown function), and *bglxA* (β-glucosidase), are essential for cellulose crystallization, and despite knowledge of their enzymatic functions, how they take part in this process is unclear (29–32).

In this report, the terms used to describe the cellulose assembly process are adapted from the ones defined in reference 29, elaborating on the cell-directed hierarchical model for cellulose crystallization (7, 10). Glucan chains are linear polymers of β-1,4-linked glucose residues synthesized by a single catalytic site of a cellulose synthase. An elementary fibril (also termed a minicrystal in previous work [10, 33, 34]) is the product of the periplasmic aggregation of multiple glucan chains which is then extruded through a single BcsC subunit into the environment. Microfibrils result from the aggregation of several elementary fibrils, at least three according to earlier work (34), outside the cell. These microfibrils can then crystallize into sheets that stack on each other to form ribbons. The latter terminology differs somewhat from previous usage in that our definition of a sheet is equivalent to the “bundles of microfibrils,” the polymerization step prior to the ribbon, described in reference 29.

Much work has already been done to understand the synthesis of paracrystalline cellulose (18, 20, 21, 23, 30–33, 35–41). In particular, freeze fracture/freeze-etching electron microscopy (EM) studies have found that the Gluconacetobacter hansenii BCS complexes are arrayed linearly along the side of the cell (18, 33, 38, 39), and this arrangement seems to determine the extracellular organization of cellulose I into ribbons (18, 33, 39). How this linear arrangement is achieved is not known.

Here, we used cryo-electron tomography (cryo-ET) of isolated cells and cryo-focused-ion-beam (cryo-FIB) milling of biofilms to visualize native cellulose production in *G. hansenii* and Gluconacetobacter xylinus, allowing the morphological characterization of the cellulose ribbons in a near-native state. We identified a novel cytoplasmic structure, which we call the cortical belt. We found that this cortical belt is absent from Escherichia coli 1094, which produces amorphous cellulose, and Agrobacterium tumefaciens, which produces crystalline microfibrils but not higher-order sheets, suggesting that the cortical belt functions to align BCS complexes to produce cellulose sheets.

## RESULTS

### Cellulose is laid out in stacked sheets on one side of the cells.

**FIG 1:**
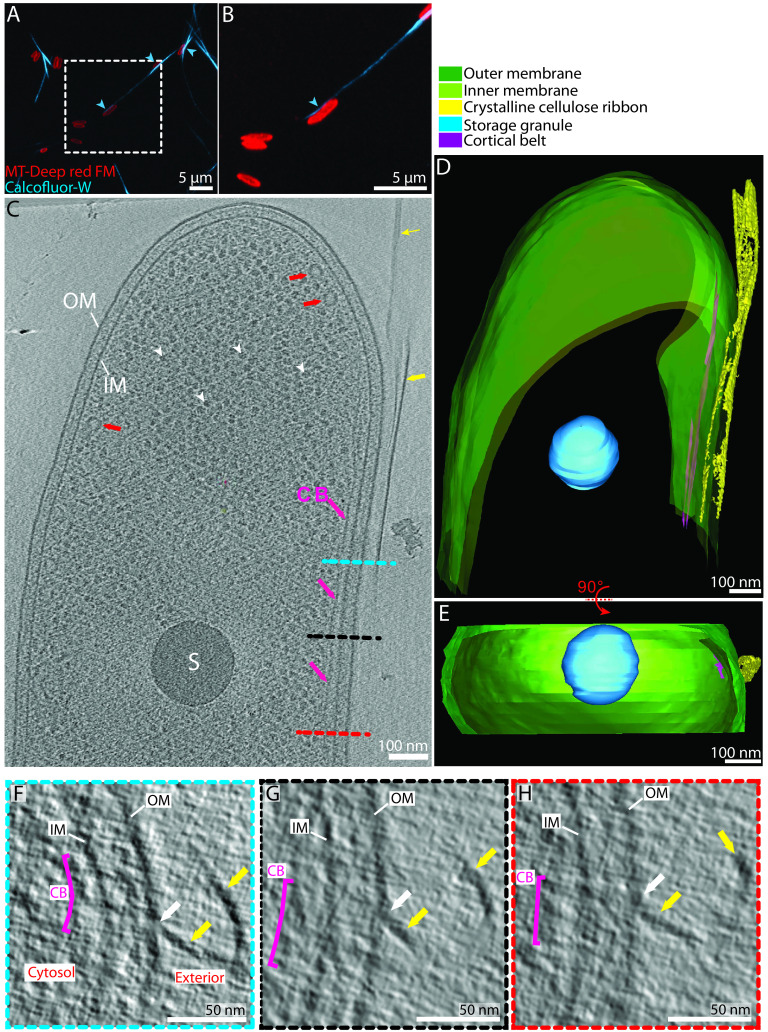
Interactions between the bacterial envelope and the cellulose ribbon: the tight configuration. (A) Confocal Airyscan optical slices show representative examples of *G. hansenii* cells in red (MitoTracker Deep Red FM) displaying the cellulose ribbon on their side in cyan (calcofluor white). (B) Enlarged view indicated by dashed rectangle in panel A. The cellulose structure is clearly seen closely appended to one side of the cell (cyan arrowheads). (C) A 9-nm-thick tomographic slice showing the typical *G. hansenii* cell harboring the cellulose ribbon on its right side (yellow arrows). White arrowheads point to ribosomes, and red arrows point to cytosolic vesicles. IM, inner membrane; OM, outer membrane; S, storage granule; CB, cortical belt. (D) Manual segmentation of the cell shown in panel C. (E) Rotated segmented volume of the image in panel D showing the very close contact between the cellulose ribbon (yellow) and the outer membrane (green). (F to H) Transverse 9-nm-thick tomographic slices through the bacterial envelope of the cell shown in panel C at the levels indicated by the blue, black and red dashed lines, respectively. Two cellulose sheets (yellow arrows) are seen. One interacts with the OM all along (white arrows). Our working model is that integration of the cellulose fibers into the sheet occurs immediately upon secretion.

To visualize bacterial cellulose production, we used cryo-ET to image intact frozen-hydrated *G. hansenii* cells separated from their cellulose biofilm according to the original method of Brown et al. (38). Previous work showed that newly synthesized cellulose ribbons are visible under the electron microscope at 1 h postseparation (38). To ensure that the cells would have enough time to synthesize cellulose ribbons, we imaged cells 5 h (300 min) after separation. To confirm cellulose production, we stained cells with MitoTracker Deep Red FM to visualize membranes and calcofluor white to visualize cellulose. By confocal imaging, we observed cellulose filamentous structures tens of micrometers long (Fig. 1A and B, cyan arrowheads). We next plunge froze cells at the same time point and imaged them by cryo-ET. The rod-shaped cells always lay flat on the grids, but their long axis was oriented randomly in the grid plane. Of 33 cells imaged, we found putative cellulose ribbons associated with 29 (88%), always on one side of the cell, including the top and bottom, and always aligned with the cell’s long axis (Fig. 1C to E, yellow arrowheads). To confirm that the ribbon was in fact cellulose, we treated cells with cellulase and observed a large reduction in the occurrence of ribbons in cryo-EM and negatively stained images (see Fig. S1, yellow arrowheads, in the supplemental material). Instead, we observed aggregated material we think is likely partially digested cellulose (Fig. S1F, orange arrowheads).

**FIG 2:**
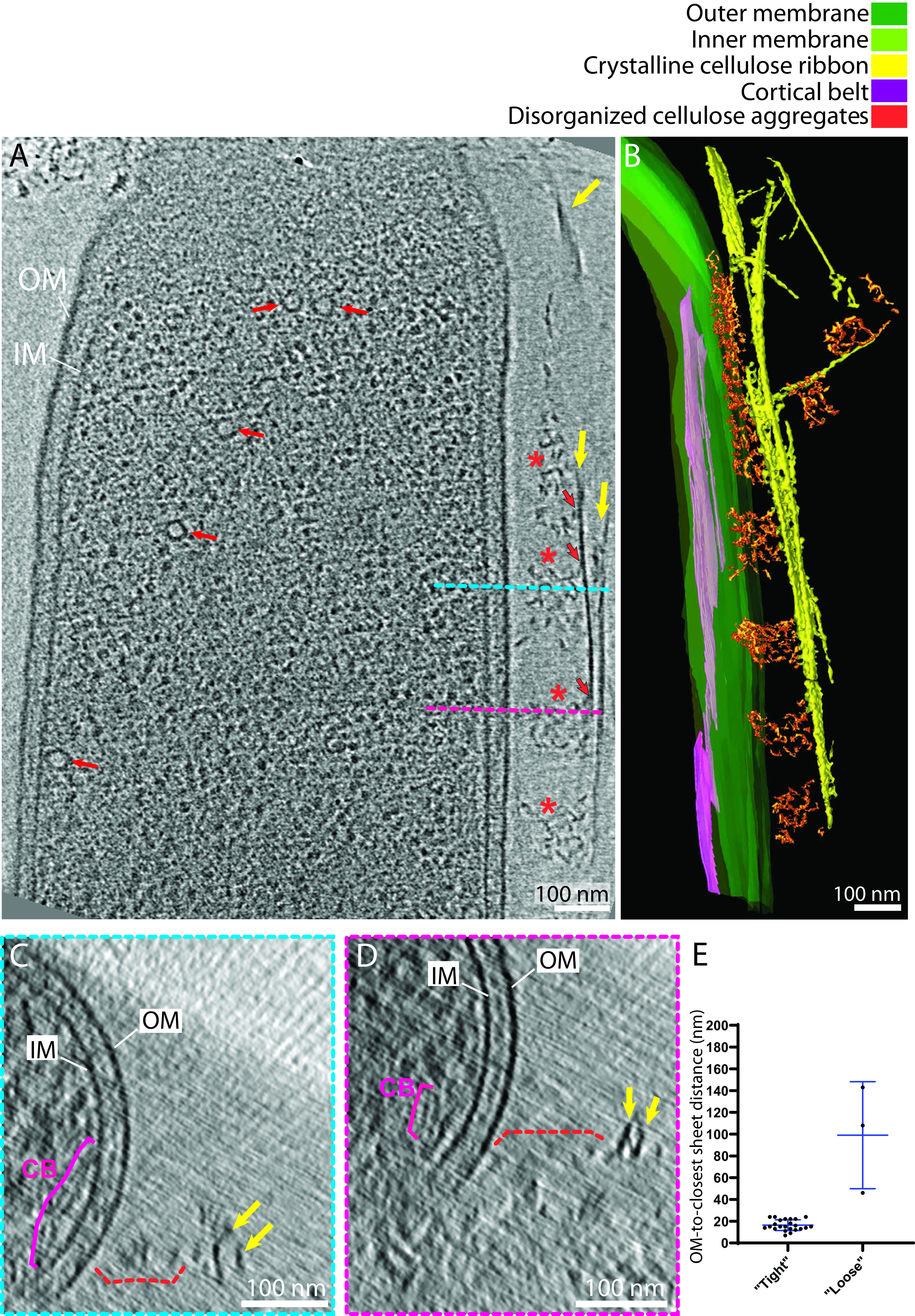
Interactions between the bacterial envelope and the cellulose ribbon: the loose configuration. (A) A 9-nm-thick tomographic slice showing a cell where aggregates of disorganized cellulose (asterisks) occur between the ribbon (yellow arrows) and the OM. Notethat the cortical belt (CB) cannot be seen in this slice. Black-outlined orange arrows indicate points of contact between the cellulose sheet and the disorganized aggregates. Red arrows point to vesicles. (B) Manual segmentation of the tomogram in panel A showing these disorganized aggregates in 3D. (C and D) Transverse 9-nm-thick tomographic slices through the envelope of the cell shown in panel A at the levels indicated by the blue and pink dashed lines highlighting the distance between the two cellulose sheets (arrows) and the OM and the presence of the disorganized clusters (dashed brackets). (E) Plot showing the OM-to-closest-sheet distance in the two types of configuration. *n* = 3 and 23 for the loose and tight configuration, respectively. Two-tailed *P* value = 0.0008 (Mann-Whitney test).

The spatial relation between the cellulose ribbons and the OM was examined. In 3 of the 29 tomograms, the cellulose ribbon was observed running beneath or on top of the cell, causing it to be normal to the electron beam; thus, it was inherently not well resolved, and it was difficult to assess its spatial relation with the OM (42). Therefore, data from these 3 tomograms were excluded for these measurements. In the remaining tomograms, two distinct configurations were observed: a tight configuration in 23 of 26 tomograms (88%), where the average OM-to-ribbon distance was 16 ± 5 nm (*n* = 23) (Fig. 1C to H; supplemental video 1 [https://figshare.com/s/74891ac625fe8125c60c]), and a loose configuration in 3 of 26 tomograms (12%), where the average OM-to-closest-sheet distance was 99 ± 49 nm (*n* = 3) (Fig. 2), most probably resulting from a mechanical stress causing the cellulose ribbon to pull away from the cell. Among the tomograms showing a tight configuration, 17 of 23 (65%) displayed multiple clear direct contacts between the OM and the ribbon (Fig. 1F to H, white arrows). Tomograms in the loose configuration exhibited ribbons that seemed detached from the OM, with an increased OM-to-closest-sheet distance compared to the tight configuration (Fig. 2E). All three tomograms presented disorganized aggregates bearing a mesh-like appearance between the OM and the ribbon (Fig. 2A to D, orange asterisks and dashed bracket). These aggregates always connected to the ribbon (Fig. 2A, black-outlined orange arrows). Throughout the study and in line with previous studies, *G. hansenii* was never seen harboring a flagellum, pili, curli, or any appendages other than the cellulose ribbons (Fig. S2) (90, 91). Additionally, similar cellulose aggregates have been seen previously by negative staining (18, 28); hence, we are confident that these structures are cellulose in a disorganized form.

**FIG 3:**
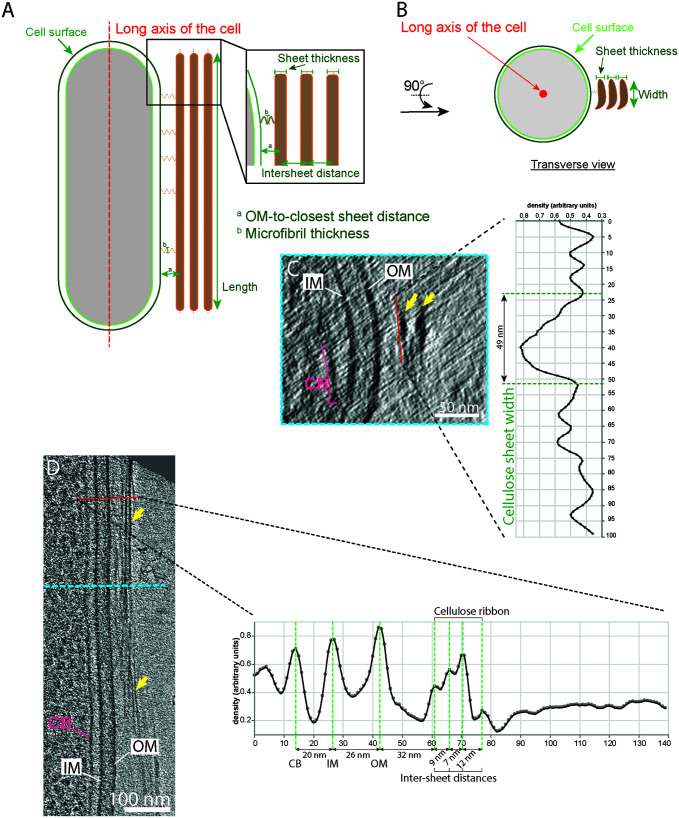
Cellulose sheet dimensions. (A and B) Longitudinal and transverse schematic depictions defining the different dimensions measured, namely, OM-to-sheet distance, sheet width, and intersheet distance. Identical terminology is used for the measurements of the cortical belt. (C) Transverse 12-nm-thick slice of the bacterial envelope of the cell shown in panel D at the level indicated by the blue dashed line. The arrows highlight the two stacked sheets. On the right, the average density profile along the red line demonstrates how the cellulose sheet widths were estimated. Vertical axis is length in nanometers along the red line and horizontal axis is the normalized electron density. (D) A 12-nm-thick tomographic slice showing the typical organization of the bacterial envelope on the side where cellulose sheets (arrows) are being synthesized. The average density profile on the right taken along the red line shows the CB-IM, IM-OM, OM-sheet and intersheet distances (green dashed lines).

These cells and their cellulose structures (the ribbons) were imaged in a near-native (frozen-hydrated) state, allowing measurement of their native dimensions. In our description of the cellulose ribbons below, “length” refers to the dimension parallel to the long axis of the cell (Fig. 3A), “thickness” refers to the dimension normal to the cell surface (Fig. 3A, enlargement), and “width” refers to the dimension tangential to the cell surface (Fig. 3B). The cellulose ribbons we observed were very similar to what has been seen previously by negative-stain EM (28, 38). Ribbons comprised long flexible stacked sheets, too long to be measured by cryo-ET because they are never entirely in the field of view. Relative to previous morphological work, our flexible sheets equate to what was described as “microfibrillar bundles” in previous studies (10, 28, 29, 34). However, our observations of them in a frozen-hydrated state allowed us to visualize them in a sheet-like configuration; therefore, we chose to call them sheets instead of bundles. Missing-wedge-induced Z-elongation of the cellulose sheets distorts width measurements (43, 44). Despite this artifact, we estimated the width at 38 ± 14 nm (*n* = 45) (Fig. 3C), which is therefore an overestimate. To see if width increased along the cell, width estimates were performed along the length of the cellulose ribbon in 3 tomograms. Unfortunately, these estimates are heavily influenced by the missing-wedge-induced elongation in the Z-dimension; therefore, the measurements did not reveal any conclusive trend in one way or another (increase, decrease, or constant width along the cellulose ribbon). These sheets then stack into a ribbon (2.3 ± 0.9 sheets on average; *n* = 24), with a variable intersheet distance (16 ± 7 nm; *n* = 23). Intersheet distance was accurately measured from peak to peak (Fig. 3D), which encompasses 2 halves of the two neighboring sheets’ density and the space between them (Fig. 3A, enlargement). Because the apparent thickness of single densities in cryo-ET is strongly affected by the defocus applied, individual cellulose sheet thickness measurements will be overestimated. Therefore, we can say confidently only that they are thinner than the intersheet distance. Despite careful inspection, although densities could be seen in the periplasmic space, we did not recognize a consistent shape which we could confidently attribute to the BCS machinery. This is likely due to the large cell diameter (∼800 nm) and the small size and/or flexibility of the BCS complexes.

### Sheets arise from the stacking of microfibrils.

**FIG 4:**
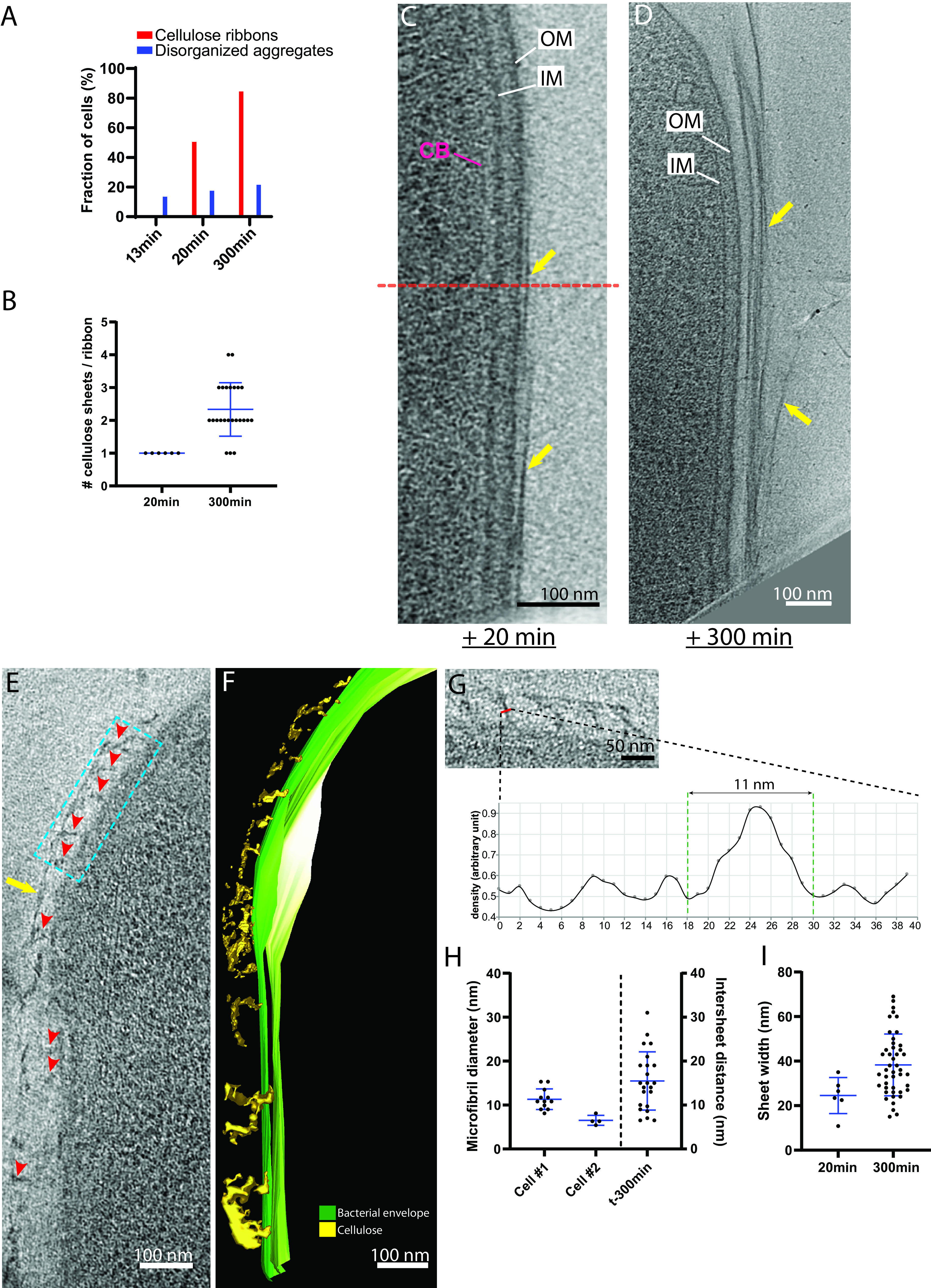
The cellulose ribbon is a composite structure made of stacked sheets. (A) Percentages of cells exhibiting disorganized aggregates (blue) and cellulose ribbons (red) at 13-, 20- and 300 min postseparation. While disorganized aggregate occurrence is steady, there is an increase in the occurrence of cellulose ribbons over time. *n* = 6, 15, and 33 for 13, 20, and 300 min, respectively. (B) Number of cellulose sheets composing the ribbons as a function of time after cell separation. *n* = 6 and *n* = 21 tomograms for 20 and 300 min postseparation,respectively. Two-tailed *P* < 0.0001 (one-sample Wilcoxon signed rank test against a theoretical value of 1 [the number of sheets observed at 20 min postseparation]). (C) Composite image, composed of 10-nm thick tomographic slices spaced by 24 nm in Z, of a cell 20 min postseparation in the tight configuration. The cellulose ribbon is thin (arrows), being composed of one sheet immediately adjacent to the OM. Limits of the two original images are indicated by the dashed line. (D) An 11-nm-thick tomographic slice of a cell 300 min postseparation. The cellulose ribbon (arrows) is large and composed of multiple sheets. (E) Nascent cellulose sheet 20 min postseparation (arrow). Putative microfibrils can be seen coming out perpendicularly from the outer membrane (arrowheads). (F) Corresponding manual segmentation of the image in panel E. (G) Enlarged view of the boxed region in panel E. Below is the average density profile showing the estimation of the diameter of one putative microfibril (red line). (H) Estimated diameters of microfibrils observed at 20 min postseparation in the two cells where they are visible (left vertical axis) as in panel E and the intersheet distances measured in the 300-min-postseparation cellulose ribbons (right vertical axis). Twelve and four microfibril thickness measurements were performed on two separate tomograms (cells 1 and 2). Forty-seven measurements for intersheet distances were performed on 23 tomograms. ANOVA followed by Tukey’s multiple-comparison test was performed. Cell 1 versus cell 2, cell 1 versus 300-min intersheet distances, and cell 2 versus 300-min intersheet distances showed adjusted *P* values of 0.073, 0.15, and 0.0015, respectively. (I) Sheet width estimations at 20 and 300 min postseparation. Six and 45 sheets were measured at 20 and 300 minutes postseparation. Welch’s *t* test (parametric *t* test without equal SD assumption) showed a *P* value of 0.23.

To visualize earlier stages of cellulose synthesis, we plunge froze cells at earlier time points after separation from the biofilm. A total of 6 and 15 tomograms were acquired at 13 and 20 min postseparation, respectively. At 13 min (the most quickly we could complete plunge freezing), no cells exhibited a cellulose ribbon; however, disorganized aggregates were observed in the vicinity of 1 of the 6 tomograms. At 20 min postseparation, cellulose ribbons were observed adjacent to the cell in 9 out of 15 tomograms (64% versus 88% [*n* = 33] at 300 min postseparation) (Fig. 4A). Of these 9 cells harboring an adjacent cellulose ribbon, 3 had it on the top or bottom of the cell and were excluded from the analysis for the reason given above. Therefore, the analysis of the OM-ribbon interface was conducted on the remaining 6. The cellulose ribbons observed at 20 min postseparation comprised only one cellulose sheet (*n* = 6), a significantly smaller amount than at 300 min postseparation (*P* < 0.0001) (Fig. 4B). Four of these 6 tomograms (67%) exhibited a tight configuration. The average OM-to-closest-sheet distance of 14 ± 3 nm (*n* = 4) was not significantly different from the 300-min postseparation tight configuration average OM-to-closest-sheet distance (*P* > 0.9; *n* = 4 and 23 for 20 min and 300 min postseparation, respectively) (Fig. 4C and D). The two other tomograms showed ribbons in the loose configuration, i.e., at an OM-to-closest-sheet distance of >40 nm, with disorganized aggregates between. These loose ribbons had OM-to-closest-sheet distances of 43 and 59 nm, respectively.

The disorganized aggregates visible at 20 min postseparation emanated perpendicularly from the OM to connect to the nascent cellulose sheet. They were thinner than the ones observed at 300 min postseparation and rod shaped (Fig. 4E and F, arrowheads). Average density profiles normal to the direction of the cylindrical-shaped densities were traced to estimate their diameter (Fig. 4G). We again emphasize the inherent overestimation of such measurements due to defocus. The average estimates on the two cells, 11 ± 2 nm (*n* = 12) and 6.5 ± 1 nm (*n* = 4) (Fig. 4G) therefore establish upper limits of the true diameter. These estimates are also less than the above-measured intersheet distances (Fig. 4H). Because elementary fibrils are thought to be between 1.5 and 6 nm in thickness (18, 38, 39), we hypothesize that these structures are microfibrils composed of several elementary fibrils. The variability of the microfibril diameter measurements between cells (Fig. 4G, cells 1 and 2) suggests that these structures can contain a varying number of elementary fibrils more or less tightly packed together. This configuration is reminiscent of what was seen in previous studies of microfibrils coming out of clusters of pores (28, 38) and likely represents an early stage of cellulose sheet formation that has been mechanically disturbed. Sheets at 20 min postseparation had an estimated width of 25 ± 8 nm (*n* = 6) (Fig. 4I), smaller than those at 300 min, although the difference did not appear significant (*P* = 0.26).

These results show that (i) the microfibrils emanating from the OM have roughly the same thickness as the cellulose sheet, (ii) sheet width seems to increase over time, and (iii) the number of cellulose sheets comprising a ribbon increases over time.

### A novel cytoplasmic structure is associated with cellulose production.

**FIG 5:**
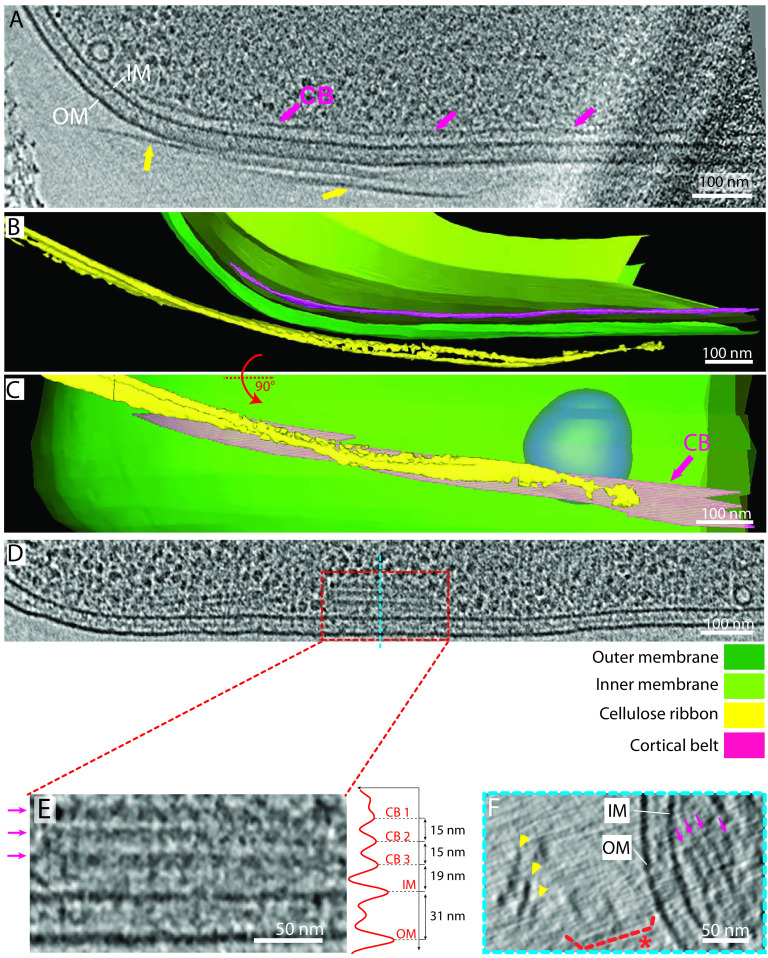
The cortical belt lies below the cellulose ribbon in the cytoplasm. (A) A 9-nm-thick tomographic slice showing a representative cortical belt (purple arrows) just inside the IM and proximal to the cellulose ribbon on the outside of the cell (yellow arrows). (B) Manual segmentation of the tomogram shown in panel A highlighting the cellulose ribbon and the cortical belt. (C) Same segmentation rotated 90° about the long axis of the cell shows how the cortical belt and the cellulose ribbon follow the same trajectory. (D) A 9-nm-thick tomographic slice taken from the same tomogram as in Fig. 2, showing one of several cases where the cortical belt presented stacked layers (dashed box). (E) Enlarged view of the boxed region in panel D showing the arrangement of the stacked layers. On the right is a density profile displayed normal to the cortical belt to measure the interlayer distance (15 nm). (F) Transverse 9-nm-thick tomographic slice of the cell region shown in panel D, at the level indicated by the blue dashed line, highlighting stacked layers of the cortical belt. The cellulose ribbon can be seen at a distance (yellow arrowheads) with disorganized aggregates in between (dashed bracket and asterisk).

We next examined the interior of *G. hansenii* cells during cellulose synthesis. These cells had extensive cytoplasmic vesicles in the center and at the periphery of the cell (Fig. S3), which is a rare and largely uncharacterized aspect of bacteria (45). The most notable feature we observed was another ribbon-like structure closely associated with the inner membrane (24 ± 4 nm from it; *n* = 19) (for an example of peak-to-peak measurement, see Fig. 3D) and several hundred nanometers in length (Fig. 5A, purple arrows). We found it in 90% of cells with a cellulose ribbon (*n* = 29), always on the same side as, and underlying, the nascent cellulose sheet (Fig. 5B and C; supplemental video 2 [https://figshare.com/s/74891ac625fe8125c60c]). This cytoplasmic structure is not a tube but rather a stack of sheet-like structures, 47 ± 23 nm wide (*n* = 10), parallel to the inner membrane and spaced (peak to peak) by 15 ± 5 nm (*n* = 7) (Fig. 5D to F). We refer to it here as the cortical belt. Interestingly, in tomograms acquired under shaking conditions in SH medium supplemented with cellulase, although the cellulose ribbons had vanished, the cortical belt was observed (Fig. S1F, purple arrows).

### Structural hallmarks of crystalline cellulose synthesis are also present in intact biofilms.

**FIG 6:**
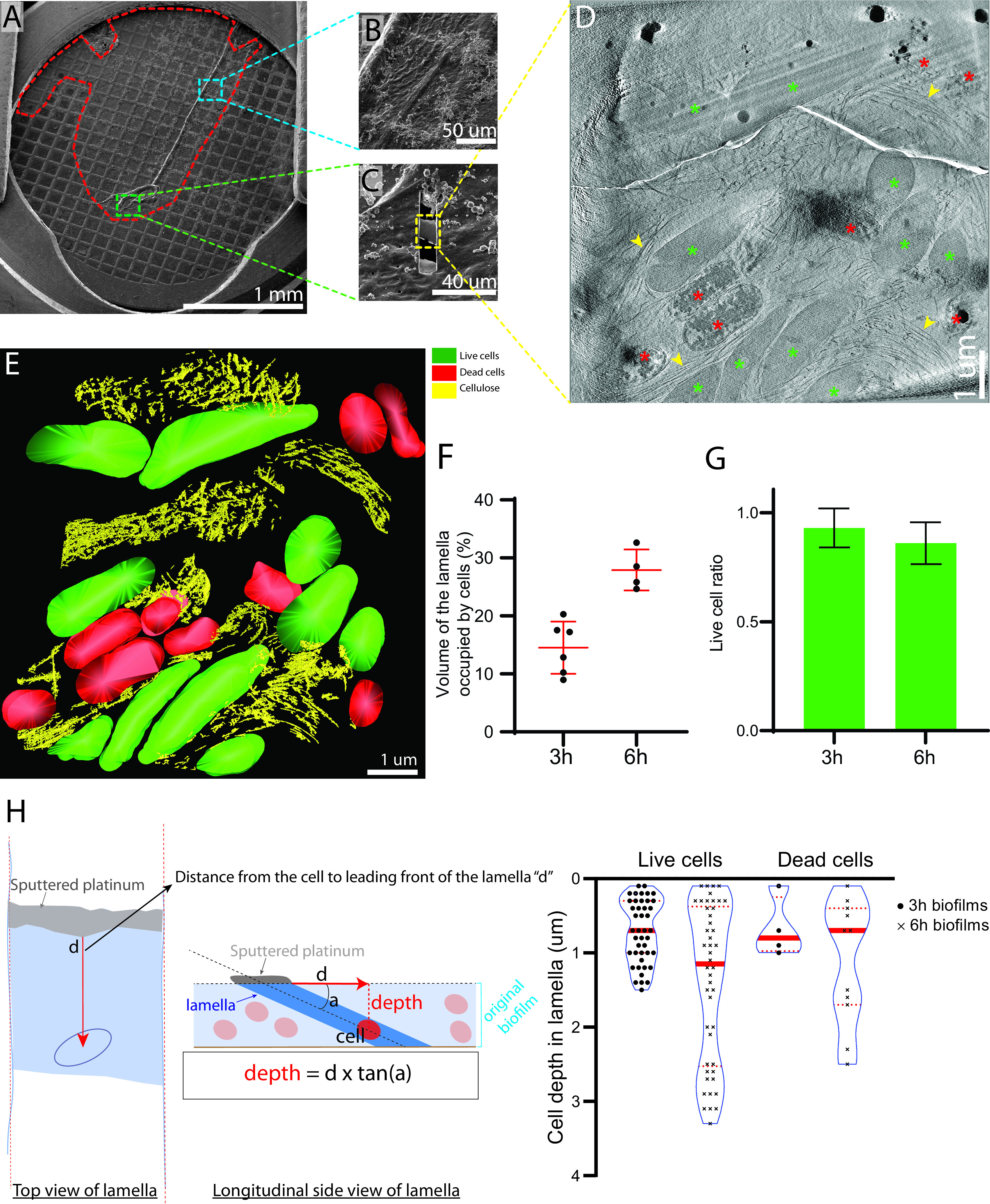
FIB milling through native *G. hansenii* biofilms. (A) Cryo-SEM overview of a 6-h biofilm (outlined in red) grown on a gold Quantifoil grid. (B) Cryo-SEM view of a thick biofilm area (boxed in blue in panel A). Wrinkles in the biofilm are typical of a biofilm a few micrometers thick. (C) Milled lamella (boxed in yellow) from the green boxed region in panel A. (D) A 23-nm-thick tomographic slice of a low-magnification tomogram of the lamella shown in panel C. Live (when frozen) and dead cells are visible (green and red asterisks, respectively), and large cellulose arrays can be seen filling the gaps between the cells (arrowheads). (E) Manual segmentation of the tomogram shown in panel D. (F) The fraction of the lamella volume occupied by the cells was assessed for each lamella. Six and 4 biofilms were grown for 3 h and 6 h, respectively. An unpaired *t* test showed a two-tailed *P* value of 0.0011. (G) Live cell ratio in 3 h and 6 h biofilms. Six and 4 biofilms were grown for 3 h and 6 h, respectively. An unpaired *t* test showed a two-tailed *P* value of 0.2720. (H) Violin box plots reporting the absolute depth of the live and dead cells within the biofilms grown for 3 and 6 h. The dashed red lines indicate the first and third quartiles, and solid red lines represent medians. This shows that while the biofilms get thicker with time, the ratio of live to dead cells appears constant through depth and time. The method of calculation is detailed on the left of the panel and in Materials and Methods. The lamella is drawn in blue, with the platinum-coated leading edge is in gray. *n* = 49, 46, 4, and 11 for live and dead cells in 3-h and 6-h biofilms, respectively. Mann-Whitney tests were performed on live versus dead cells in 3-h and 6-h biofilms, showing two-tailed *P* values of 0.82 and 0.54, respectively.

It is possible that separating bacteria from the cellulose mat for whole-cell cryo-ET imaging could have altered structures associated with cellulose synthesis. We therefore imaged *G. hansenii* cells *in situ* in young cellulose biofilms grown on gold grids. We imaged biofilms after 3 or 6 h before plunge freezing in the hope of visualizing any change in the ordering of the fibers or the aspect of the cells over the course of biofilm growth. To access cells within the 5- to 10-μm-thick biofilm, we used cryo-FIB milling to generate thin (∼200-nm) lamellae suitable for imaging by cryo-ET (Fig. 6A to C). In a total of 19 analyzed tomograms (9 and 10 tomograms for 6-h and 3-h biofilms, respectively) (Table 1), we observed fields of living and dead bacteria encased in a matrix of bundled cellulose ribbons at both time points (Fig. 6D and E; supplemental video 3 [https://figshare.com/s/74891ac625fe8125c60c]). Overview tomograms (low magnification with low total dose) and high-resolution composite images of the lamellae allowed extraction of positional information of the cells in relation to the biofilm. There were 0.10 ± 0.02 and 0.27 ± 0.04 cell/μm (2), and 15% and 28% of the volume of the lamellae was occupied by cells at 3- and 6-h time points, respectively (Fig. 6F) (*n* = 6 and 4 lamellae, respectively). This approximate 2-fold increase in cell density from a 3-h to a 6-h biofilm suggests that cell division occurs during biofilm growth.

**TABLE 1:** Tomography results^*a*^

Imaging and species or strain	Condition or strain	No. of lamellae	No. of tomograms	Source
Whole-cell tomography				
*G. hansenii* ATCC 23769	Untreated; 13 min postseparation	NA	6	ATCC
	Untreated; 20 min postseparation	NA	14	
	Untreated; 300 min postseparation	NA	33 (24 VPP)	
	Cellulase, 0.2 g/liter	NA	4	
*G. xylinus* ATCC 700178	Untreated; 5 h postseparation	NA	8	ATCC
E. coli 1094	Non-cellulose induced	NA	1	Gift from Jean Marc Ghigo (Institute Pasteur)
A. tumefaciens C58^*b*^	WT	NA	47	Gift from Patricia Zambrisky (UC Berkeley) to E. I. Tocheva
	A139	NA	10	Gift from Patricia Zambrisky (UC Berkeley) to E. I. Tocheva
	AD348	NA	1	Gift from Anath Das (University of Minnesota) to D. Ghosal
	AD1484	NA	1	Gift from Anath Das (University of Minnesota) to D. Ghosal
	JX148	NA	4	Gift from Patricia Zambrisky (UC Berkeley) to E. I. Tocheva
	NT1	NA	2	Gift from Patricia Zambrisky (UC Berkeley) to E. I. Tocheva

Tomography on milled lamellae				
*G. hansenii* ATCC 23769	Native biofilm; untreated	10	19 (3 VPP)	ATCC
E. coli 1094 induced forcellulose synthesis	Untreated	2	6	Gift from Jean Marc Ghigo (Institut Pasteur)
	Cellulase, 0.2 g/liter	1	2	

a VPP, number of the tomograms in which the Volta phase plate was used; NA, nonapplicable; UC, University of California.

b Strains were imaged for other purposes but used here as well. C58 (ATCC 33970) is wild-type A. tumefaciens. All A. tumefaciens strains listed had a C58 background.

Dead cells can be easily differentiated from living cells (Fig. 6D, red asterisks) by the wavy aspect of their envelope, sometimes presenting punctures, and by the appearance of their cytosol. Living cells typically have ribosome-rich and nonribosomal regions (bacterial chromosome), while dead cells have coagulated cytosols with large electron-dense aggregates and very low ribosome counts. The live-to-dead-cell ratio was calculated at 0.9 ± 0.1 in both 3- and 6-h biofilms, revealing no increase in the proportion of dead cells between these two time points (Fig. 6G). Because lamellae give access to the native organization and layering of the cells within the biofilm, the depth of dead/living cells within the biofilm was assessed by measuring their distance from the leading edge of the lamella (see Materials and Methods). No trend between cell depth within the biofilm and state of the cells was detected (Fig. 6H).

**FIG 7:**
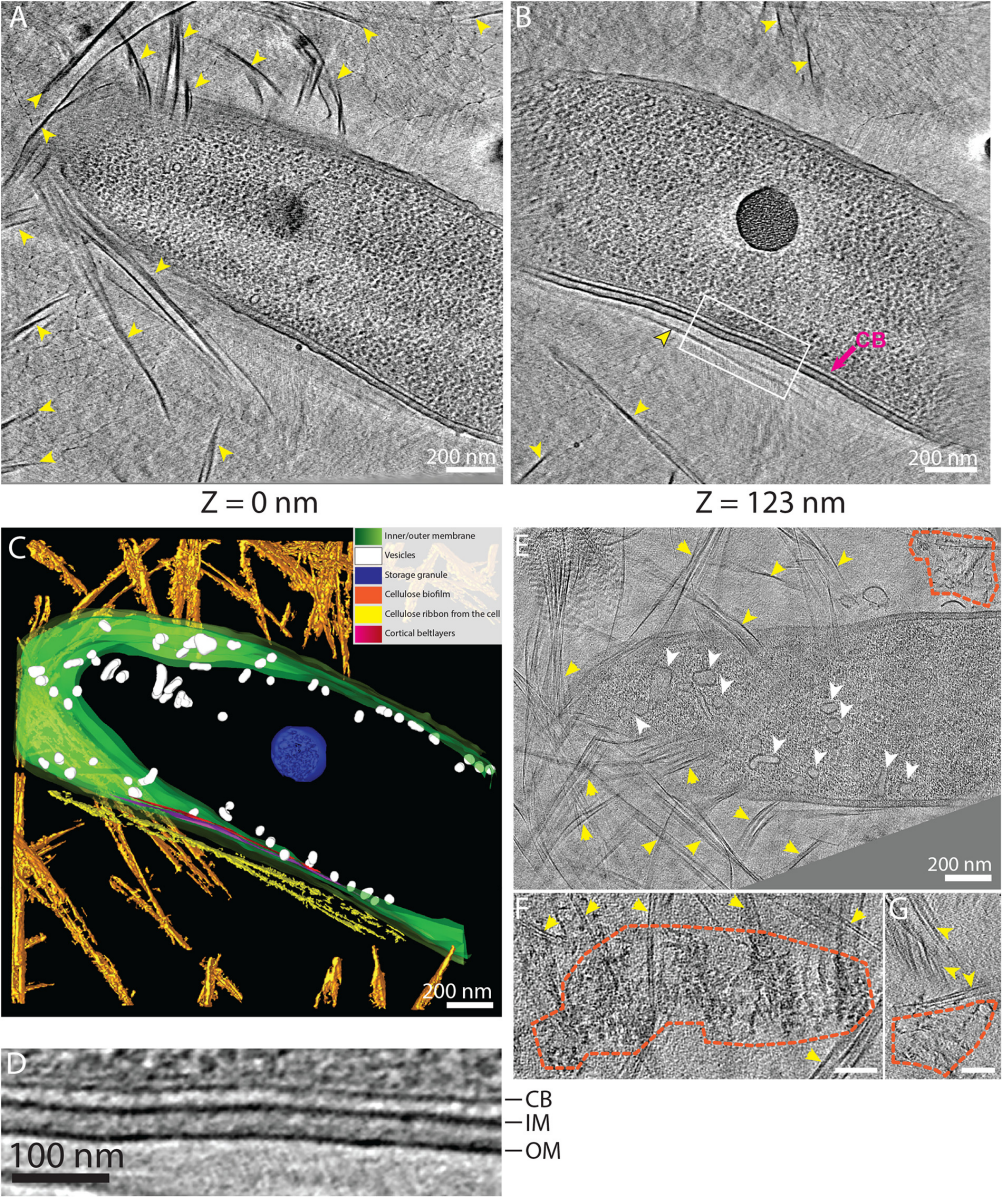
Lamellae of native biofilms also reveal numerous vesicles and the cortical belt. (A and B) Two tomographic slices of a *G. hansenii* cell from a biofilm grown for 6 h surrounded by cellulose ribbons (yellow arrowheads). The cortical belt is visible in panel B (arrow) and seems to follow the trajectory of the cellulose sheet proximal to the OM (dark-outlined yellow arrowhead). (C) Manual segmentation of the tomogram displayed in panels A and B showing the juxtaposition of the cortical belt (purple to red) and the nascent cellulose ribbon (yellow). (D) Enlargement of the boxed region in panel B showing the layered cortical belt. (E) Tomographic slice of a cell surrounded by cellulose ribbons (yellow arrowheads) from a biofilm grown for 3 h and harboring numerous vesicles in its cytosol (white arrowheads). Disorganized aggregates (dashed lines) are visible at this time point. (F and G) Tomographic slices showing additional examples of disorganized cellulose aggregates (dashed lines) surrounded by cellulose ribbons (arrowheads) visible in 3-h biofilms. Bars, 100 nm. All tomographic slices are 11 nm thick.

In all 19 tomograms (combining 3-h and 6-h lamellae), we observed numerous cellulose ribbons surrounding the cells (Fig. 7A, yellow arrowheads). In 5 of the 19 tomograms (26%), a cellulose ribbon was closely appended to the cell’s OM, as we had seen in separated cells (Fig. 7B and C, dark-outlined yellow arrowhead). Among those 5 tomograms, 4 showed a cortical belt adjacent to the cellulose ribbon (Fig. 7B to D; supplemental video 3 [https://figshare.com/s/74891ac625fe8125c60c]). The OM-to-cellulose ribbon distance (19.2 ± 8 nm; *n* = 4) and inner-membrane-to-cortical-belt distance (22 ± 2 nm; *n* = 4) were very similar to those measured in separated cells. In 5 of the 10 tomograms in 3-h biofilm lamellae, disorganized cellulose aggregates were observed connected to well-ordered ribbons just as in the separated cells, whereas this was never observed in the 6-h biofilms. This suggests that crystallization is disrupted more often in early biofilm growth (Fig. 7E to G, dashed outlining). Because *Gluconacetobacter* cells are thick, electron transmittance in the central region of the cytoplasm is very low when whole cells are imaged, making it difficult to visualize this area. Reducing sample thickness to approximately 200 nm by cryo-FIB milling allowed us to observe these central regions with greater contrast and visualize the extensive vesicle network deep inside the cell (Fig. 7E, white arrowheads) while losing the ability to capture the full extent of the cellulose sheet stacking because of the lamellar sampling.

### The cortical belt is specific to bacterial species that produce crystalline cellulose ribbons.

To see whether the cortical belt is specific to *G. hansenii*, we imaged another species of *Gluconacetobacter*, *G. xylinus* (also referred to as Komagataeibacter sucrofermentans BPR-2001), by cryo-ET at 300 min postseparation. *G. xylinus* (originally called Acetobacter xylinum) is a species isolated from cherry, bearing the ability to produce an increased amount of cellulose under shaking culture conditions (35). *G. hansenii* and *G. xylinus* have diverged quite substantially and differ in their GC content, and *G. hansenii* has its *bcsA* and *bcsB* genes fused and harbors no gene clusters associated with acetan metabolism, which are commonly found in other *Gluconacetobacter* species (46). In our hands, we also observed that *G. xylinus* biofilms seem to grow more slowly and are stiffer than *G. hansenii* biofilms. Four of 8 cells (50%) exhibited an extracellular cellulose ribbon along the cells’ long axis (Fig. S4A). The cellulose ribbons observed had 2 sheets of cellulose, with an estimated average width of 27 ± 16 nm (*n* = 5). All four cells also possessed a cortical belt (Fig. S4A and B, purple arrows), with dimensions similar to those in *G. hansenii*. The average distance from the cortical belt to the inner membrane was 24 ± 4 nm (*n* = 4). In one instance, the cortical belt also contained three stacked layers spaced (peak to peak) by 9 nm (Fig. S4C).

**FIG 8:**
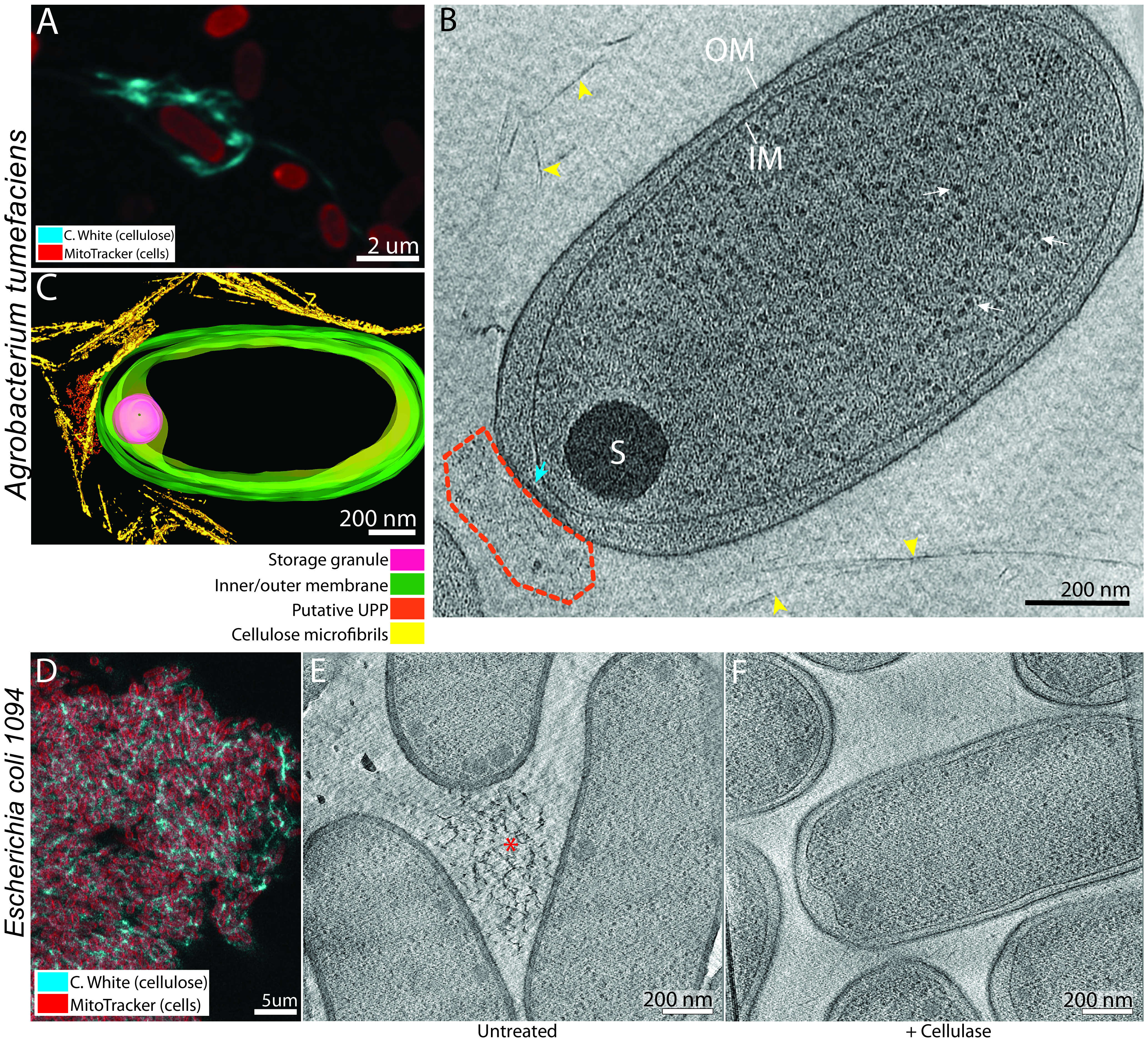
The cortical belt is not found in other cellulose-synthesizing species. (A) Maximum projection of A. tumefaciens cells synthesizing cellulose. Cells are stained with MitoTracker Deep Red (red) and cellulose with calcofluor white (cyan). (B) A 10-nm-thick tomographic slice of a typical A. tumefaciens cell with cellulose microfibrils around (arrowheads). No cortical belt can be seen in the cells. A polar flattening can be seen at the lower pole (cyan arrow) with an amorphous aggregate (dashed lines). These aggregates are most probably the unipolar polysaccharide (UPP) synthesized specifically by A. tumefaciens. (C) Manual segmentation of the tomogram in panel B showing the organization of the cellulose microfibrils around the cell, the absence of the cortical belt, and the putative UPP. (D) A 50-nm optical slice of an induced E. coli 1094 cellulose biofilm. Cells are stained with MitoTracker Deep Red (red) and cellulose with calcofluor white (cyan). (E) A 6-nm tomographic slice of a lamellar tomogram of a bacterial mat showing three E. coli 1094 cells and an amorphous cellulose aggregate between them (asterisk). (F) A 6-nm tomographic slice of a lamella through a bacterial mat treated with cellulase, showing multiple cells. No cellulose was visible under this condition. No cortical belt can be seen in the cells under either condition.

Aside from *Gluconacetobacter*, other bacterial species produce different types of cellulose. For instance, Escherichia coli 1094 can make amorphous cellulose (47), and Agrobacterium tumefaciens makes paracrystalline cellulose microfibrils during plant infection (48). Neither of these species is known to make cellulose ribbons, though. We asked whether structures similar to the cortical belt observed in *Gluconacetobacter* were present in these species. Our lab had previously imaged A. tumefaciens for other studies, and therefore cryo-tomograms of A. tumefaciens were already available. We confirmed by confocal microscopy that A. tumefaciens produces cellulose under the growth conditions used for the earlier experiments (Fig. 8A) and then screened the available tomograms for the presence of cellulose. As the purpose of the previous studies was not cellulose synthesis observation, relatively few (65 of 1,854 tomograms) showed distinct cellulose fibers in the vicinity of the cells (Fig. 8B and C, arrowheads; supplemental video 4 [https://figshare.com/s/74891ac625fe8125c60c]). These fibers did not adopt any preferential orientation and ran in all directions around the cell. They also had a smaller width (14 ± 5 nm; 52 fibers measured in 5 tomograms) than *G. hansenii* cellulose sheets (*P* < 0.0001), confirming that A. tumefaciens does not elaborate wide cellulose sheets or ribbons but rather produces simpler structures of crystalline cellulose, presumably bundles of microfibrils. In the 65 cellulose-producing cells, we never observed a cortical belt structure. However, two notable features were observed: (i) a polar outer membrane flattening in 28 cells with a thickening of the OM (43% of the 65 cells presenting cellulose) (Fig. 8B, cyan arrow) and (ii) polar amorphous aggregates in 24 cells (37% of the 65 cells presenting cellulose) (Fig. 8B, dashed outlining). Nineteen cells exhibited all three described features: the polar flattening, the amorphous aggregates, and the cellulose fibers. We suspect that these polar amorphous aggregates are the unipolar polysaccharides (UPP) described in previous work and shown to allow the attachment of A. tumefaciens to biotic and abiotic surfaces in the early stages of biofilm formation (49). The very close proximity of the putative UPP to the polar flattening suggests that the latter could hold the UPP-secreting complexes.

We confirmed that Escherichia coli 1094 grown in minimal medium produces cellulose (Fig. 8D). The cells aggregated, making it difficult to image single cells by cryo-ET, so instead we FIB milled through bacterial mats, producing ∼200-nm-thick lamellae. To identify cellulose structures, we also imaged lamellae from cultures grown in minimal medium supplemented with cellulase. In 3 of the 5 tomograms of untreated cells, we observed amorphous fibrous material (Fig. 8E, asterisk) that was not visible in 2 tomograms of a cellulase-treated culture (Fig. 8F). None of the cells imaged under either condition contained a cortical belt (13 untreated and 5 cellulase-treated cells), suggesting that it is unique to bacteria producing higher-order paracrystalline cellulose structures, i.e., sheets.

## DISCUSSION

Here, we characterized bacterial cellulose synthesis in two *Gluconacetobacter* species and compared it to that in two other species by cryo-ET. We identified a novel cytoplasmic structure associated with the production of cellulose I ribbons in *Gluconacetobacter* spp. We also performed cryo-FIB milling followed by cryo-ET on native biofilms.

### Cryo-ET confirms the need for a tight interaction between the nascent sheet and the OM.

The cell-directed hierarchical model proposes linearly arranged 3.5-nm-diameter pores on the surface of the cell (39), each extruding an elementary fibril (28, 33). The arrangement of these pores in lines allows the crystallization of the elementary fibrils upon secretion and integration into a cellulose sheet parallel to the long axis of the cell (7, 50, 51). Our results agree with this model. Indeed, we observed that when the gap between the nascent sheet and the OM exceeds approximately 40 nm, disorganized aggregates occur (Fig. 2). In keeping with previous work that observed similar events (28), we hypothesize that these aggregates are microfibrils failing to integrate into an ordered sheet. Furthermore, it has been shown that the addition of compounds which bind directly to cellulose drastically alters the assembly of the sheets and leads to the formation of similar aggregates (18, 28, 51). It appears as though preventing the nascent microfibrils from interacting with each other upon secretion prevents them from forming one organized sheet. Conversely, a confined spacing between the nascent sheet and the OM promotes proper crystallization of the nascent microfibrils. This proximity could be maintained either by a previously synthesized sheet preventing the nascent one from separating too far from the OM or by specialized cellulose binding enzymes situated in the outer leaflet of the OM, such as CmcAx, which has the ability to bind cellulose (52).

### Cryo-ET sheds light on the buildup of a microfibril.

Many values have been reported for the elementary fibrils’ dimensions, mainly through direct observation by negative-staining electron microscopy (18, 33, 38). The most favored hypothesis is an approximately 1.5-nm-thick elementary fibril (thoroughly discussed in reference 28). Very recently, the characterization of the structure of the BcsC subunit (the OM pore) described a 1.5-nm-inner-diameter pore with a very narrow constriction caused by a mobile gating loop, restricting the channel to a 0.2-nm bottleneck (24). It is, however, not known to what extent this gating loop can open the pore. Therefore, two hypotheses arise: (i) one BcsC pore can accommodate a 1.5-nm elementary fibril through an opening of the gating loop, or (ii) it can accommodate a smaller elementary fibril, perhaps only a single glucan chain. In the latter case, the building of the elementary fibril would then take place upon secretion of the glucan chains in the environment.

While negative staining has provided high-resolution views of cellulose ribbons (28, 38), observing them in a frozen-hydrated state enables more accurate measurements of their dimensions and observation of their interaction with the OM. This is particularly important for extracellular polysaccharides, which have been shown to collapse and undergo drastic conformational changes upon dehydration, staining, and embedding (53).

**FIG 9:**
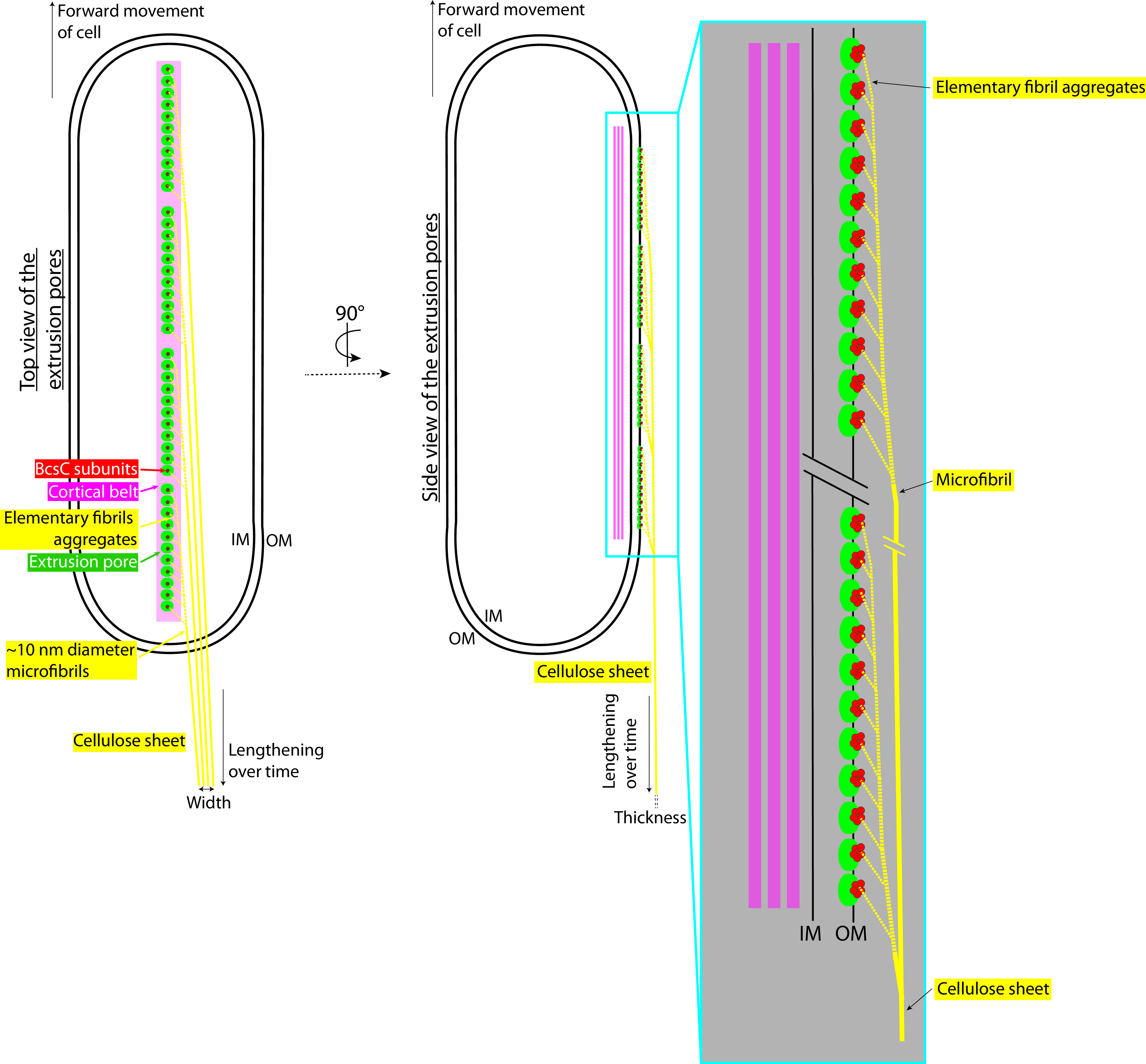
Updated cell-directed hierarchical model. Top (left) and side (right) views of a *G. hansenii* cell showing the different aggregation steps leading to a cellulose sheet, how microfibrils contribute to sheet width, and the role of the cortical belt. In this model, clusters of 11 extrusion pores are depicted (green circles); the real numbers and distribution are unknown. Each extrusion pore is presented as comprising 5 BcsC subunits (red circles); the actual number is not known. On the right is a magnified view of the line of 11 extrusion pores, each hypothesized to extrude an aggregate of multiple elementary fibrils (yellow dashed lines). All aggregates then coalesce to form a microfibril of increasing thickness as it incorporates an increasing number of elementary fibril aggregates. These microfibrils then stack together, contributing to the width of the cellulose sheet. Adapted from the cell shown in Fig. 4E to G.

We were able to image, in two tomograms, microfibrils extruded perpendicularly to the OM and integrating to form a thin parallel sheet (Fig. 4E and F). A possible explanation of why these events are rare is that they result from an accidental mechanical separation of the nascent sheet from the OM, revealing early forms of cellulose bundling, such as thin microfibrils. As explained above, precise measurement of the thickness of densities is difficult in cryo-ET, since it is influenced by the defocus applied during imaging (causing overestimation of the true thickness). Despite this uncertainty, our measurements are done in a near-native state. We estimated these microfibrils to be less than 11 nm in diameter (Fig. 4G and H), in line with previous work which measured microfibril thicknesses from 3 to 12 nm in cellulose sheets splayed apart by cellulase treatments (54). If we assume that an elementary fibril is 1.5 nm in diameter and that it can go through a single BcsC subunit, an 11-nm-diameter cylindrical microfibril (maximal thickness estimation) would comprise 53 elementary fibrils. This would require a cluster of 53 BcsC subunits. Previous reports have stated that the cellulose extrusion pores cluster in linear bunches of 2 to 4 pores (7, 33). Accommodating both observations would require that there be more than one BcsC subunit per extrusion pore. For example, if each 3.5-nm-diameter extrusion pore (39) maximally held 5 BcsC subunits, a cluster of 11 extrusion pores could produce an 11-nm-diameter microfibril (Fig. 9). In this case, each extrusion pore holding multiple BcsC subunits would produce a crystalline aggregate of elementary fibrils which would pack with its neighboring aggregates to form a microfibril.

### Cryo-ET sheds light on the assembly of a cellulose sheet.

We found that ribbons were stacks of sheets that likely interact loosely with one another, since the intersheet distance varied from 7 to 31 nm. This loose stacking corroborates previous observations (34). Previous measurements done by negative staining had estimated cellulose sheet width to range from 40 to 600 nm (28, 38, 54), wider than our measurements, which ranged from 11 to 69 nm (Fig. 4I). These variations have been attributed to the cell strain, growth conditions, and intercellular variation (28, 38, 39). We found that the thickness of cellulose sheets is similar to the diameter of the microfibrils. Therefore, our data suggest that microfibrils lie in rows to create the width of the sheet. This was also suggested in reference 7.

While the number of sheets produced by a single cell increased with time, the main dimension of growth appears to be ribbon length, as suggested by previous work and our fluorescence data showing cellulose ribbons several cell lengths long (Fig. 1A and B) (29). Wider sheets occur at later time points (Fig. 4I), suggesting that sheet width also grows with time. However, in the current model, sheet width is correlated with the number of extrusion pores, and hence with cell length (7, 39). It is possible that at 300 min postseparation, cells are longer and possess more extrusion pores, therefore producing wider sheets. As mentioned earlier, our attempt to observe this by monitoring sheet width along its length failed. The magnification employed to acquire the data would allow us to capture only partial lengths of the cells and their cellulose ribbon. We think that for the segments we captured, the increase or decrease in sheet width, probably in the range of 10 to 20 nm, was unlikely to be observed because of the estimates being heavily influenced by the missing wedge.

### Cryo-ET on *G. hansenii* cells allowed the visualization of a novel cytoskeletal element, the cortical belt.

Negative-stain, cryofracture, and immuno-EM studies have shown that cellulose extrusion pores in *Gluconacetobacter* align in a line on one side of the cell (28, 39, 55), but what causes this alignment is unknown. Here, we identify a novel cytoplasmic structure in two species of *Gluconacetobacter* that spatially correlates with the nascent cellulose ribbon (Fig. 1C to E and Fig. 5). This structure, which we term the cortical belt, is found at a fixed distance from the inner membrane (24 ± 4 nm) and remains intact upon cellulase treatment under shaking conditions (see Fig. S1F, purple arrow, in the supplemental material), suggesting that it is stable even in the absence of the cellulose ribbon and under turbulent culture conditions.

We observed the cortical belt in both *Gluconacetobacter* spp. imaged but not in other bacteria that produce less-ordered forms of cellulose, including Escherichia coli 1094, which synthesizes amorphous cellulose (47), and Agrobacterium tumefaciens, which synthesizes cellulose I microfibrils (56) (Fig. 8). This suggests that the cortical belt is a peculiar cytoskeletal filament found only in the genus *Gluconacetobacter*. Its striking spatial colocalization with the extracellular cellulose ribbons leads us to propose that the cortical belt functions in the formation of cellulose ribbons. The periplasmic BcsD and its interacting partner CcpAx (22, 31), as well as two cell wall-related enzymes, have been shown to be involved in the crystallization process of the ribbons (29, 31, 57). It is possible that the cortical belt interacts with one or more of these components to guide the positioning of the BCS complexes. Unfortunately, as stated earlier, we failed to identify any repeated density above the cortical sheet that could be associated with the secreting complexes. However, the thick cells and crowded periplasm obscured and very likely masked relevant densities. Moreover, it is unknown whether these secreting complexes are channels spanning the periplasmic space, given the structures of the individual components (19–21, 24, 58). Their predicted position in the membranes shows very small portions protruding in the periplasm. We therefore think that the BCS complexes are too small/flexible for particle picking and subtomogram averaging in such a crowded environment. If the cortical belt is responsible for scaffolding the BCS complexes, it represents a novel prokaryotic cytoskeletal element, i.e., “a cytoplasmic protein filament and its associated superstructures that move or scaffold material within the cell” (59). Other bacterial cytoskeletal elements have been observed to form belt-like structures, including bactofilins (60), or to stack, like the CTP synthase (61), although with different dimensions. We hope that future work will identify the component(s) that forms the cortical belt, shedding more light on the molecular processes involved in the organization and clustering of the BCS complexes in *G. hansenii*.

### The cortical belt reveals another similarity between cellulose synthesis in *Gluconacetobacter* and land plants.

Historically, the first plant cellulose synthase genes were identified by cDNA homology with the *G. xylinum acsA* (*bcsA*) gene (62). Later on, phylogenetic studies highlighted an early divergence between cyanobacterial and plant cellulose synthases (63, 64). A large number of cellulose I-synthesizing organisms have in common that the synthase complexes are arranged in specific patterns, determining the final architecture of the cellulose structures (7). A simple row is formed in systems like *Gluconacetobacter* spp. or certain charophytes and chlorophytes (65), and hexameric rosette structures called cellulose synthase complexes (CSC) are found in land plants. In both, the extrusion of a crystalline form of cellulose exerts a force believed to be able to propel the CSCs in plants (66, 67) and the whole cell in *Gluconacetobacter* (29, 38). Our work uncovers an additional similarity, the involvement of a cytoskeletal element, the cortical belt, to guide the synthase complexes. In land plants, CSCs have been shown to interact indirectly with underlying cortical microtubules, mediating transmembrane cross talk (68–70) and guiding and regulating CSC velocity (71–73). While CSCs were shown to be motile in land plants, they are believed to be static in *Gluconacetobacter* (28), perhaps held in place by the cortical belt, in order to transfer the propelling force to the whole cell.

### Insights from FIB milling of native biofilms.

Cryo-FIB milling through native biofilms offers the possibility of observing bacteria in the context of their original biofilm environment and retrieving high-resolution morphological and positional information about the cells relative to one another and relative to the biofilm layers. Visualization of the density and organization of the extracellular matrix and its interaction with the cells is also rendered possible by cryo-FIB milling. This is especially important since in nature, most bacterial species are found in complex interacting communities, in the form of homogeneous or heterogeneous communities that organize in biofilms (14).

Milling the *Gluconacetobacter* biofilms to 200 nm revealed numerous cytosolic vesicles of variable shapes and sizes. Although we were not able to connect the presence of these numerous vesicles with the process of cellulose production, cytosolic vesicles in bacteria are uncommon but have already been observed several times in Myxococcus xanthus, A. tumefaciens, and E. coli, for example (45). Their detailed structure, function, and biogenesis are not known. The cortical belt was also visible, as in the isolated cells. The cellulose ribbons aligned with each other to form larger arrays 2 to 3 μm wide (Fig. 6D, arrowheads; supplemental video 3 [https://figshare.com/s/74891ac625fe8125c60c]), showing the propensity of these structures to interact with each other. This propensity was previously characterized by live imaging of the cellulose biosynthesis and crystallization process in *Gluconacetobacter*, which showed that the bacterial cells preferentially follow already established tracks, i.e., previously synthesized cellulose ribbons (29). The occurrence of disorganized cellulose clusters in biofilms grown for 3 h but not 6 h suggests that such aggregates are either (i) digested by enzymes, likely CmcAx, reported to have an endoglucanase capable of digesting amorphous cellulose (74) and to be present on the surface of *G. hansenii* or released in the environment (30, 52) or (ii) diluted by a gradual increase in well-ordered ribbons over time.

Cell death in biofilms, with the fraction of dead cells measured at 10% in our biofilms, is a well-known phenomenon (14), caused by programmed cell death mechanisms, cannibalistic behaviors such as those described for Bacillus subtilis (75), or nutrient/oxygen depletion (76, 77). We did not observe a preferential location of dead cells at the bottom of the biofilm, ruling out anoxic conditions being the primary cause of cell death. This could be because the thickness of the biofilm, between 1.5 and 3 μm according to the cell depth distribution (Fig. 6H), is too small to have a significant oxygen gradient, as suggested by studies that determined that total anoxia is generally reached between a 70- and 80-μm depth (77–79). Processing biofilms that are thicker (i.e., in the range of tens of micrometers) would allow visualization of the effects of nutrient/oxygen gradients on cell distribution and physiology. For now, plunge freezing, as was performed in this study, can properly vitrify only samples that are less than ∼10 μm thick (80). Moreover, milling thicknesses above 8 to 10 μm becomes labor-intensive and technically difficult. A possible course of action for further studies would be to perform high-pressure freezing on thicker biofilms and then produce thin sections either by cryosectioning, by hybrid cryosectioning/FIB milling methods such as those described in references [Bibr B81][Bibr B82] 83, or by a cryo-lift-out procedure (83).

## MATERIALS AND METHODS

### Cell culture.

Gluconacetobacter hansenii (ATCC 23769) was cultured as previously described (37) in SH medium: 2% glucose, 0.5% Bacto peptone, 0.5% yeast extract (pH 6). For solid medium, 2.5% Bacto agar was added. Cells were separated from the cellulose biofilm by mechanical disruption as previously described (38). Briefly, the bacterial cellulose biofilm developing at the air-medium interface was picked up with a single-use sterile inoculating loop and transferred to fresh medium, where it was vigorously shaken and then removed. In preparation for freezing, cells were pelleted by centrifugation for 10 min at a relative centrifugal force (RCF) of 2,500 at 20°C and resuspended in 0.5 ml of SH medium. The culture was incubated for the desired length of time at 30°C without shaking before plunge freezing. For cellulose digestion, 0.2 g/liter cellulase (purified exo- and endoglucanases; number LS002598; Worthington) was added.

Gluconacetobacter xylinus (ATCC 700178/BPR2001) was cultured as described above in fructose-peptone-yeast extract (FPY) medium: 2% fructose, 1% Bacto peptone, 0.5% yeast extract, and 0.25% K_2_HPO_4_.

Escherichia coli 1094 was cultured in lysogeny broth (LB) and induced for cellulose production in minimal medium: 0.2% (NH_4_)_2_SO_4_, 1.4% KH_2_PO_4_, 0.1% MgSO_4_, 0.5% FeSO_4_·7H_2_O, 0.4% glucose, 0.01% thiamine (pH 7). A saturated overnight LB culture was diluted 1:50 in 3 ml of minimal medium with or without 0.2 g/liter cellulase (purified exo- and endo-glucanases; number LS002598; Worthington). Cultures were incubated at 37°C with shaking at 220 rpm. When the medium transitioned from turbid to clear and white flakes appeared (cellulose and bacteria), the induction of cellulose synthesis was considered successful.

Agrobacterium tumefaciens was cultured as described in previous work (84). Briefly, A. tumefaciens C58 was cultivated in liquid AB medium (0.2% glucose, 18.7 mM NH_4_Cl, 2.5 μM MgSO_4_, 2 mM KCl, 0.07 mM CaCl_2_, 0.01 mM FeSO_4_, 8.4 mM K_2_HPO_4_, 4.16 mM NaH_2_PO_4_·7H_2_O [pH 7]) at 30°C overnight. Induction was done by pipetting 100 μl of overnight culture and spreading it onto AB induction plates (0.2% glucose, 18.7 mM NH_4_Cl, 2.5 μM MgSO_4_, 2 mM KCl, 0.07 mM CaCl_2_, 0.01 mM FeSO_4_, 8.4 mM K_2_HPO_4_, 4.16 mM NaH_2_PO_4_.7H_2_O, 1.7% Bacto agar, 100 μM acetosyringone [pH 5.8]). Plates were then incubated for 3 days at 20°C. Cells were resolubilized by scraping a small amount from the plate with an inoculation loop and resuspending it in 100 μl of liquid induction AB medium.

The following strains were included in the tomogram analysis. NT1 is a C58 strain without plasmid pTiC58 (tumor inducing). A139 is NT1REB(pJK270) + pJZ041. NT1REB is a “bald” strain, i.e., a no-flagellin mutant, derived from NT1. The plasmid pJK270 is pTiC58 with the transposed NPTII gene for kanamycin resistance. Plasmid pJZ041 carries a green fluorescent protein (GFP)-tagged VirB8 gene, encoding a component of the type IV secretion system (T4SS) (85). Strain JX148 is a C58-derived mutant with a mutation of the *rem* gene. The strain is nonmotile. AD348 is a GV3101(pMP90) strain with its whole VirB system deleted. GV3101 is a pTiC58-free, rifampin-resistant C58 strain, and pMP90 is a helper pTiC58 without the T-DNA. AD1484 is an AD348 variant, transformed with pAD2079 containing the whole VirB system.

### Confocal microscopy.

Cellulose was stained with calcofluor white (number 18909; Sigma-Aldrich) at a concentration of 0.001%, and cell membranes were stained with MitoTracker Deep Red FM (number M22426; Thermo Fisher) at a concentration of 0.5 μg/μl. Stack acquisition was done on a Zeiss LSM880 Airyscan microscope. Airyscan acquisitions were performed in superresolution mode with the Z-step set at the optimal optical sectioning. The Mito-Tracker Deep Red FM channel was set as follows: excitation at 633 nm, use of the 488/561/633 main beam splitter, and a band-pass 570–620 + long-pass 645 filter. The calcofluor white channel was set as follows: excitation at 405 nm, use of the 405 main beam splitter, and a band-pass 420–480 + band-pass 495–550 filter. Airyscan processing was performed on the fly by the in-built algorithm of Zeiss Black.

### Sample preparation for cryo-EM.

For isolated cells, Quantifoil Cu R2/2 Finder grids (Quantifoil Micro Tools GmbH) were glow discharged at 15 mA for 1 min. The grids were preincubated with fiducial marker solution prepared as follows: 50 μl of 10 nm colloidal gold (Ted Pella, Inc.) was mixed with 50 μl of 5% bovine serum albumin (BSA), vortexed for 1 min, and centrifuged at an RCF of 15,000 for 15 min; the supernatant was discarded, and the pellet was resuspended in 40 μl of phosphate-buffered saline (PBS) buffer. A 3-μl sample was deposited on each grid, left for 1 min, and then back-blotted with Whatman paper. Cells were plunge frozen with a Vitrobot Mark IV (Thermo Fisher Scientific) with 100% humidity at 30°C and back-blotted for 3 to 5 s.

For native biofilms, Quantifoil gold R2/2 Finder grids were placed in 35-mm glass-bottom petri dishes (number P35G-1.0-2.0C; MatTek Corporation) containing 1 ml of SH medium inoculated with a 2-day-old biofilm. The dishes were sealed with Micropore tape (3M) and incubated without shaking at 30°C for 3 to 6 h. Plunge freezing was done at 22C and 50% humidity, either with manual blotting on both sides of the grids (first back-blotted and then front-blotted) or by using the automatic blotting function of the Vitrobot with a blotting time of 5 to 6 s, a blotting force of 15, and a drain time of 2 s.

For E. coli 1094, after 4 h of incubation in minimal medium, the medium should turn from turbid to clear with white flakes. The optical density at 600 nm (OD_600_) of the cultures was monitored using the culture (always turbid) where cellulose induction was performed in the presence of cellulase to keep the cells from aggregating. It was then used as a reference to concentrate the cells to a high OD_600_ (10 to 20), in order to form bacterial mats on the EM grids, for control and cellulase conditions. Plunge freezing was done at 20°C and 100%, either with manual back-blotting for 5 to 7 s and a drain time of 1 s or by using the automatic blotting function of the Vitrobot with a wait time of 10 s, a blotting time of 5 to 6 s, a blotting force of 3, and a drain time of 1 s.

### FIB milling.

Grids were clipped in Autogrid holders (Thermo Fisher) machined with a notch to allow FIB milling closer to the edge of the grid. Autogrids were placed in a custom-built shuttle and inserted into a Versa 3D dual-beam FIB/SEM microscope with a field emission gun (FEG) (FEI) equipped with a PP3000T cryo-transfer apparatus (Quorum Technologies). They were maintained at −175°C at all times by a custom-built cryo-stage (86). To reduce sample charging and protect the sample from curtaining during milling, the grids were sputter coated with platinum at 15 mA for 60 s. Thin lamellae were generated with the Ga^+^ ion beam at 30 kV at angles ranging from 10 to 17°. Rough milling was done at high currents, ranging from 0.3 nA to 100 pA, until the lamellae measured 1 μm in thickness under the FIB view. Current was then progressively brought down to 10 pA for the final milling steps until the measured thickness was between 100 and 200 nm. Final polishing of the back end of the lamella was also done at 10 pA, where the sample was tilted +0.5 to 1° to homogenize the lamella thickness. During the whole procedure, imaging with the SEM beam was done at 5 kV and 13 pA.

### Electron cryotomography.

Tomography of whole cells and FIB-milled lamellae was performed on either a Titan Krios or Tecnai G2 Polara transmission electron microscope (Thermo Fisher) equipped with a 300-kV field emission gun, energy filter (Gatan), and K2 or K3 Summit direct electron detector (Gatan). The Krios microscope is equipped with a Volta phase plate (Thermo Fisher) (87). Tilt-series acquisition was done with SerialEM (88) with a 2 to 3° tilt increment for a total range of ±60° or ±50°, a defocus of −4, −6, or −8 μm, and a total dose up to 180 e^−^/Å^2^. Volta phase plate images are shown in Fig. 1, 2, 5, and 7A and B with a defocus of −2 μm and a measured phase shift of 0.5 π/rad before tilt series acquisitions.

Low-magnification tomography on the biofilm lamellae was performed at a magnification of ×6,500 (14-Å^2^ pixel size) with a −10 or −15 μm defocus and a total dose between 5 and 10 e^−^/Å^2^.

Tomography of FIB-milled lamellae was done exclusively on the Titan Krios instrument. Because samples were thinner, the total dose was limited to ∼80 e^−^/Å^2^.

### Data processing.

Tomograms were reconstructed using the IMOD software (http://bio3d.colorado.edu/imod/) (89). Alignment was done on 1,000-by-1,000 binned tilt series with fiducial-marker-based alignment. Aligned stacks were low-pass filtered (0.35, σ = 0.05) to eliminate high-frequency noise. Weighted back projection reconstruction was performed, and the SIRT-like filter was used with 20 iterations.

Segmentation was also done using IMOD and drawing tools developed by Andrew Noske (http://www.andrewnoske.com/student/imod.php). To better distinguish features during the segmentation steps, tomograms were filtered with the three-dimensional (3D) nonlinear anisotropic diffusion filter in IMOD. The cell contours and cortical belt were segmented manually on a Cintiq 21uX tablet (Wacom), and cellulose was segmented using a semiautomated thresholded method. (i) A denoising nonlinear anisotropic diffusion filter was applied (included in the etomo package [http://bio3d.colorado.edu/imod/]) on the tomogram; (ii) precise boundary models were drawn around the structures to be thresholded; (iii) thresholding segmentation was performed with 3Dmod using the isosurface function, and the previously drawn contours were used as a mask. When the contours are precisely following the features of interest, this technique makes it possible to raise the isosurface threshold without picking up background noise.

Measurements for all distances between elements (cellulose sheet to outer membrane, width of the cellulose ribbon, cortical belt to inner membrane) were taken by generating normalized density profile plots and measuring the distances between the density peaks of the corresponding subcellular features (Fig. 3). This was automated with a custom script, sideview-profile-average, written by Davi Ortega (https://www.npmjs.com/package/sideview-profile-average).

Estimation of the cell depth in the native biofilm lamellae was calculated as follows: (i) using the two parallel walls of the milled trench, a perpendicular line was traced at the leading edge of the lamella (where the platinum meets the frozen material); (ii) lines were drawn from the center of the cells to the leading edge perpendicular line (Fig. 6H, red line in top view of lamella); (iii) the distance from the cell center to the limit of the platinum on the leading edge, which is the surface of the sample, was measured. The real depth was then calculated using the following equation: opposite side (real depth) = tan(*a*) × adjacent side (distance measured [d in Fig. 6H]), where *a* is the angle between the grid surface and the FIB gun during the milling process, which can be accurately measured during reconstruction with 3dmod.

### Statistical analysis.

All statistics were performed with GraphPad Prism software (https://www.graphpad.com/scientific-software/prism/). All data sets were first analyzed for normality using the Shapiro-Wilk test and homoscedasticity (equal standard deviations). If a data set was normal, appropriate parametric tests were performed, and if not, appropriate nonparametric tests were performed. Detailed statistical tests are listed in order of appearance in the paper.

For Fig. 2E, *n* was 3 and 23 for the loose and tight configurations, respectively. Two-tailed *P* was 0.0008 (Mann-Whitney test).

For the OM-to-closest-sheet distances in cells at 20 min versus 300 min postseparation, *n* values were 4, 2, 23, and 3 for 20-min tight, 20-min loose, 300-min tight, and 300-min loose configurations, respectively. A Kruskal-Wallis test followed by Dunn’s multiple-comparison test was performed. The values for 20-min tight versus 20-min loose, 300-min tight, and 300-min loose showed adjusted *P* values of 0.12, >0.99, and 0.024, respectively. The comparisons of 20-min loose versus 300-min tight and 300-min loose and for 300-min tight versus 300-min loose showed adjusted *P* values of 0.23, >0.99, and 0.032, respectively.

For Fig. 4A, *n* values were 6, 15, and 33 for 13, 20, and 300 min, respectively. For Fig. 4B, there were 6 and 21 tomograms for 20 and 300 min postseparation, respectively. The two-tailed *P* value was <0.0001 (one-sample Wilcoxon signed rank test against a theoretical value of 1 [the number of sheets observed at 20 min postseparation]). For Fig. 4H, 12 and 4 microfibril thickness measurements were performed on two separate tomograms (cells 1 and 2, left side of the graph). There were 47 measurements for intersheet distances performed on 23 tomograms. Analysis of variance (ANOVA) followed by Tukey’s multiple-comparison test was performed. Cell 1 versus cell 2, cell 1 versus 300-min intersheet distances, and cell 2 versus 300-min intersheet distances showed adjusted *P* values of 0.073, 0.15, and 0.0015, respectively. For Fig. 4I, 6 and 45 sheets were measured at 20 and 300 min postseparation. Welch’s *t* test (parametric *t* test without equal standard deviation [SD] assumption) showed a *P* value of 0.23.

For Fig. 6F, 6 and 4 biofilms were grown for 3 h and 6 h, respectively. An unpaired *t* test showed a two-tailed *P* value of 0.0011. For Fig. 6G, 6 and 4 biofilms were grown for 3 h and 6 h, respectively. An unpaired *t* test showed a two-tailed *P* value of 0.2720. For Fig. 6H, *n* values were 49, 46, 4, and 11 for live and dead cells in 3 h and 6 h biofilms, respectively. Mann-Whitney tests were performed on live versus dead cells in 3-h and 6-h biofilms, showing two-tailed *P* values of 0.82 and 0.54, respectively.

For *G. hansenii* cellulose sheet width versus A. tumefaciens cellulose fibril width, 52, 45, and 6 width measurements were taken for A. tumefaciens and for *G. hansenii* at 20 min and 300 min postseparation, respectively. Kruskal-Wallis one-way analysis of variance followed by Dunn’s multiple-comparison test was performed. A *t* test for 20 min versus 300 min, 20 min versus A. tumefaciens, and 300 min versus A. tumefaciens showed adjusted *P* values of 0.25, 0.11, and <0.0001, respectively.

## Supplementary Material

Supplemental file 1
